# Effect of Ultrasonic Treatment on Chemical Stripping Behavior of Aluminum Coating on K6509 Co-Based Superalloy

**DOI:** 10.3390/ma18173979

**Published:** 2025-08-25

**Authors:** Yuanyuan Jin, Cheng Xie, Ke Sun, Zehuan Li, Xin Wang, Xin Ma, Hui Wang, Rongrong Shang, Xuxian Zhou, Yidi Li, Yunping Li

**Affiliations:** 1 State Key Lab for Powder Metallurgy, Central South University, Changsha 410083, China; jinyuanyuan83@csu.edu.cn (Y.J.); youxianboy@163.com (C.X.);; 2 Aero Engine Corporation of China, South Industry Co., Ltd., Zhuzhou 412002, China; 3 Madrid Institute for Advanced Studies of Materials (IMDEA) Materials Institute, C/Eric Kandel 2, Getafe, 28906 Madrid, Spain; 4 Department of Materials Science, Polytechnic University of Madrid/Universidad Politécnica de Madrid, E.T.S. de Ingenieros de Caminos, 28040 Madrid, Spain

**Keywords:** Co-based high-temperature alloy, aluminum coating, corrosion removal, ultrasonic, substrate protection

## Abstract

In this study, 10% nitric acid was employed to remove the aluminum coating on the cobalt-based superalloy K6509, with a focus on elucidating the corrosion mechanism and evaluating the effect of ultrasonic on the removal process. The results shows that ultrasonic treatment (40 kHz) significantly improves coating removal efficiency, increasing the maximum corrosion rate by 46.49% from 2.5413 × 10^−7^ g·s^−1^·mm^−2^ to 4.7488 × 10^−7^ g·s^−1^·mm^−2^ and reducing removal time from 10 min to 6 min. This enhancement is attributed to cavitation effect of ultrasonic bubbles and the shockwave-accelerated ion diffusion, which together facilitate more efficient coating degradation and results in a smoother surface. In terms of corrosion behavior, the difference in phase composition between the outer layer and the interdiffusion zone (IDZ) plays a decisive role. The outer layer is primarily composed of β-(Co,Ni)Al phase, which is thermodynamically less stable in acidic environments and thus readily dissolves in 10% HNO_3_. In contrast, the IDZ mainly consists of Cr_23_C_6_, which exhibit high chemical stability and a strong tendency to passivate. These characteristics render the IDZ highly resistant to nitric acid attack, thereby forming a protective barrier that limits acid penetration and helps maintain the integrity of the substrate.

## 1. Introduction

Cobalt (Co)- and nickel (Ni)-based superalloys have been widely used in fabrication of critical components in aerospace engines, such as turbine blades, combustion chambers, and turbine disks because of its excellent high-temperature mechanical properties and environmental resistance [1]. Compared to Ni-based alloys, Co-based superalloys demonstrate superior performance in several aspects, including higher melting points (1320–1360 °C), excellent high-temperature strength retention, and enhanced corrosion resistant originated from the high chromium content (20–30 wt.%) [2,3,4].

However, under extreme serving conditions such as high-temperature, high-pressure, centrifugal forces, aerodynamic loads, and impact loads [5,6,7], fatigue cracks, wear, and corrosion frequently occur on blades after long-term cyclic loading [8,9], which seriously threaten the reliability of aeroengines. To extend the service life of the turbine blades, surface coating with excellent mechanical and chemical compatibility are widely used. These include aluminide coatings [10,11,12], MCrAlY coatings [13,14,15], and thermal barrier coatings [16,17].

Aluminum coatings [18,19] have emerged as the predominant surface protection technologies for turbine blades due to their mature manufacturing processes and exceptional oxidation and corrosion resistance. Their protective mechanism primarily relies on the consumption of CoAl and NiAl to form a continuous Al_2_O_3_, which serves as a barrier to isolate the substrate from the oxidizing environment, significantly enhancing the component’s resistance to high-temperature degradation [20]. During service, aluminum atoms in the coating continuously diffuse outward to form a protective Al_2_O_3_ scale, while inward diffusion into the substrate causes the decomposition of the β-NiAl phase [21]. Guo et al. [22] demonstrated that such degradation in aluminum coatings is mainly driven by interdiffusion between the coating and the substrate, initiating preferentially at the coating surface, sub-layer interfaces, and the coating–substrate interface. Additionally, thermal cycling stress generated from the mismatch of coefficient of thermal expansion (CTE) between the coating and the substrate [23,24], combined with molten salt hot corrosion as well as foreign particle impact, can lead to coating degradation, cracking, and even spalling, ultimately resulting in the loss of protective functionality [25,26]. Given that the manufacturing cost of blades is significantly higher than that of the coating, premature blade retirement due to coating failure would result in substantial material and economic waste [27]. Thus, the development of reliable coating remanufacturing technologies is imperative to extend blade service life and enhance resource utilization efficiency.

Prior to coating remanufacturing, the complete removal of initial coatings from the substrate surface is a critical prerequisite to ensure the quality of recoating. Currently, industrial coating removal technologies primarily involve mechanical, chemical, and electrochemical methods [28]. Mechanical removal techniques, such as abrasive blasting, mechanical grinding, polishing, high-pressure water jet [29], and laser cleaning [30,31], utilize energy-driven mechanisms to delaminate coatings. Salinas et al. [32] conducted a systematic investigation on the influence of key abrasive water jet (AWJ) parameters (including water pressure, traverse speed, and standoff distance) on the propensity of abrasive embedding and surface topography. Their experimental results demonstrated the efficacy of pure water jets in removing residual abrasive particles. While mechanical coating removal techniques offer advantages such as strong controllability and absence of chemical contamination, they also present limitations including low efficiency, tendency to induce microcracks and residual stresses in substrate materials, challenges in achieving complete coating removal, and high equipment costs, which significantly constrain their industrial application [33].

Electrochemical coating removal enables selective stripping of surface coatings through controlled electrolytic reactions, offering advantages in process controllability and geometric adaptability [28,34,35]. However, its industrial application remains limited due to complex electrolyte management and narrow process windows, particularly when dealing with components with inconsistent surface conditions that have variable surface degradation. As a result, most electrochemical coating degradation technologies remain at the laboratory research stage, making large-scale industrial production difficult to achieve.

Compared to mechanical and electrochemical methods, chemical methods are more suitable for large-scale removal of coatings from the substrate for precision components with complex shapes, such as turbine blades [36,37,38]. Via precisely controlling process parameters (solution composition, temperature, and reaction time), chemical methods can achieve effective coating removal while minimizing damage to the substrate. For example, Alam et al. [39] achieved controlled and uniform removal of PtAl coatings on Ni-based superalloys through optimizing the HCl and H_3_PO_4_ ratio. Nevertheless, chemical methods still face limitations such as complex solution systems and prolonged treatment times. Moreover, most studies have focused on removing aluminum coatings from Ni-based alloys, with relatively little research addressing Co-based superalloys.

Currently, the removal of aluminide coatings is mainly conducted using HNO_3_- or HCl-based etching systems [28,35]. However, the presence of Cl^-^ in the HCl system significantly increases the risk of pitting corrosion of the substrate [38]. The previous study of Bouesteix et al. [40] confirmed that the use of 10% HNO_3_ solution through a 2 h chemical etching process can successfully completely remove the aluminum coating from the austenitic substrate. Wang et al. [41] employed a 10% HNO_3_ solution and successfully achieved complete removal of the NiCoCrAlY coating via electrochemical etching. Based on the above research findings, this study employs 10% HNO_3_ to remove the initial coating from the substrate surface. Particular attention was given to the role of ultrasonic assistance in the removal process, focusing on its ability to enhance coating dissolution, accelerate corrosion reactions, and promote more uniform and efficient stripping. The corrosion mechanism of the coating in acidic conditions was also analyzed to clarify the synergistic effects between chemical and ultrasonic actions. This work aims to provide a feasible and controllable approach for coating removal in the remanufacturing of high-temperature components.

## 2. Materials and Methods

K6509 alloy with dimensions of 15 × 10 × 4 mm^3^ was used in this study, and its chemical composition is listed in Table 1. A through-hole (Φ2.5 mm) was drilled on the 15 × 10 mm^2^ surface of each sample to facilitate suspension during processing, with polytetrafluoroethylene (PTFE) rope employed during corrosion testing. Prior to vapor aluminizing, the K6509 substrate was subjected to surface pretreatment by sandblasting. The process was performed using 200–240 mesh corundum grit at a pressure below 0.25 MPa and continued until a uniformly gray-white activated layer was achieved across the entire surface.

The aluminizing agent contained 30 wt.% pure aluminum powder (aluminum source), alumina (filler), and ammonium chloride (activator). Samples were heated to 980 °C at 5 °C/min and held for 8 h under flowing argon, facilitating deposition and inward diffusion of aluminum. After aluminizing, the samples were furnace-cooled to 600 °C and then air cooled to room temperature. Subsequently, ultrasonic cleaning was conducted in deionized water at 60–80 °C to remove residual surface contaminants.

**Figure 1:**
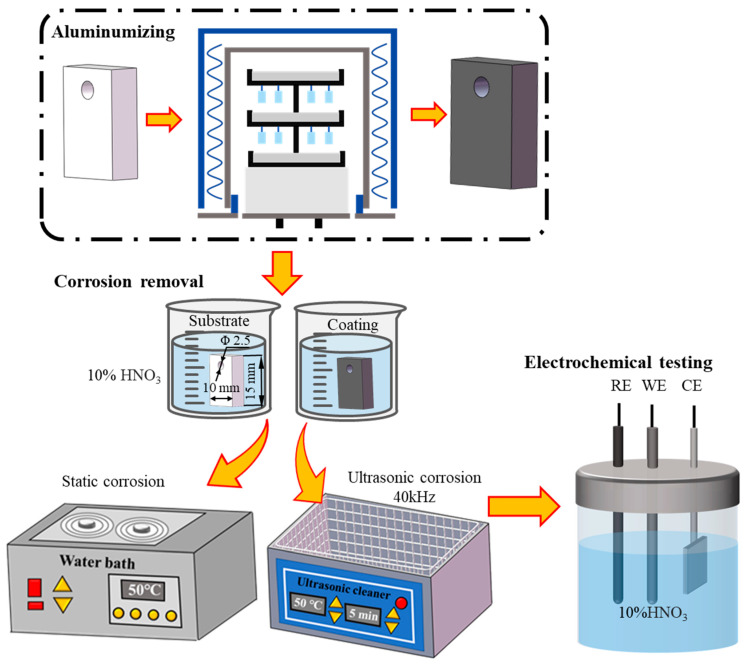
Flow chart for aluminum coating preparation and removal process.

The corrosion behavior of the Co-based superalloy K6509 was evaluated by immersing both coated and uncoated samples in a 10% HNO_3_ solution at 50 °C under ultrasonic and non-ultrasonic conditions. The ultrasonic treatment was conducted at a fixed frequency of 40 kHz. Samples were immersed for predetermined time intervals and subsequently removed from the solution. Each sample was then thoroughly rinsed with deionized water, followed by ethanol, and dried. The mass loss was measured using a high-precision analytical balance (ATY124, Shimadzu, Kyoto, Japan; resolution: 0.01 mg) to assess the corrosion rate. The detailed experiment flow chart is illustrated in Figure 1. The corrosion rate and weight loss were calculated using the following Equations (1) and (2):

(1) $$v=\frac{m_{i}-m_{i-1}}{\mathrm{ST}}$$

(2) $$\Delta G=\frac{m_{i}-m_{0}}{S}$$

where $m_{0}$ is the initial weight, $m_{i}$ and $m_{i-1}$ are the weights before and after corrosion, respectively, *S* is the surface area exposed to the solution, and *T* is the corrosion time. The corrosion process was deemed to have terminated once the corrosion rate fell below 1.0 × 10^−8^ g·s^−1^·mm^−2^, indicating a negligible rate of material loss.

Electrochemical measurements were performed using a three-electrode system configuration connected to an RST5000 electrochemical workstation (Shiruisi RST5200F Instrument, Zhengzhou, China) in a 10% HNO_3_ solution at room temperature. The sample was employed as the working electrode, a Pt sheet (10 × 10 × 0.1 mm^3^) was employed as the counter electrode, and a saturated calomel electrode (SCE) was employed as the reference electrode. The open circuit potential (OCP) was recorded for 600 s in 10% HNO_3_. The potentiodynamic polarization (PDP) scans were performed at a scan rate of 0.005 V/s within a potential range of ±0.5 V versus OCP. Electrochemical impedance spectroscope (EIS) was carried out at OCP with a frequency ranging from 10^4^ Hz to 0.01 Hz at amplitude of 0.007 V. All measurements were performed three times to ensure reproducibility.

The surfaces and the cross-sectional morphologies of the samples were characterized by a scanning electron microscopy (SEM, Quanta650, FEI, Hillsboro, OR, USA). The corresponding chemical compositions of the samples were analyzed by an energy-dispersive spectroscopy (EDS) detector. The determination of the dominant phases formed on the surface of the coating samples were detected by X-ray diffraction (XRD, Rigaku Model D/MAX 2550, Rigaku-Corporation, Tokyo, Japan). The XRD analyses were performed using Cu-Kα radiation with a wavelength of 1.54056 A° over 2θ range of 25° to 85°. Atomic force microscopy (AFM) and scanning Kelvin probe force microscopy (SKPFM) measurements were performed using a scanning probe microscope (BRUKER Dimension Icon, Siegsdorf, Germany), with a scan area of 20 × 20 μm^2^. Surface potential mapping was obtained via a dual-scan method: the first scan was conducted to collect surface topology data, while in the second scan, the tip was lifted to 128 nm above the surface to avoid interference from surface topography on the Volta potential. Both topographic and surface potential images were acquired at room temperature and 25% humidity. AFM and SKPFM data were analyzed using NanoScope Analysis 3.00 software.

## 3. Results

### 3.1. Initial Microstructure of Aluminum Coating

**Figure 2:**
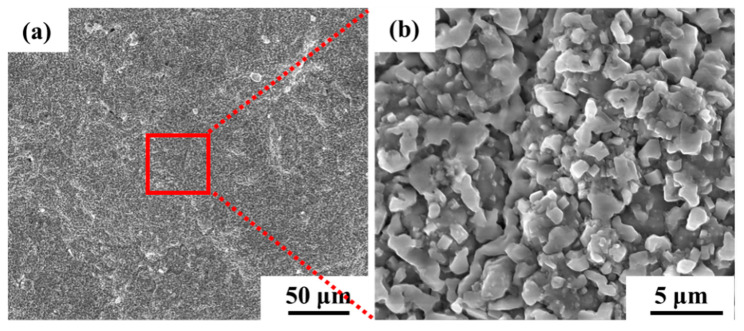
Scanning electron microscopy (SEM) morphology under secondary electrons (SE) mode of the initial aluminum coating of the Co-based superalloy: (**a**) low-magnification, and (**b**) high-magnification.

**Figure 3:**
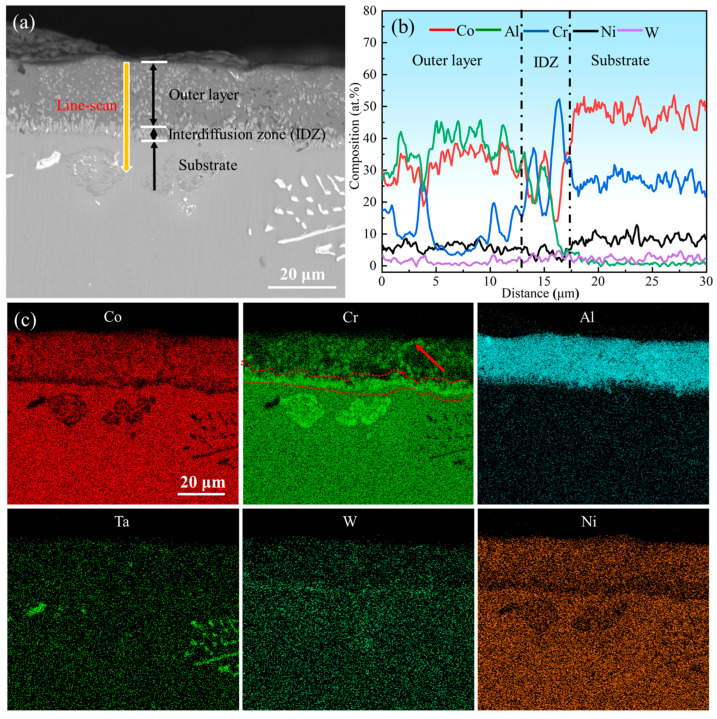
(**a**) Backscatter electron (BSE) images of the cross-section topography of the initial aluminum coating, (**b**) corresponding Energy Dispersive Spectroscopy (EDS) line-scan results marked by the yellow line in (**a**), and (**c**) corresponding EDS mapping results.

The surface morphology of the aluminum coating sample, as shown in Figure 2, revealed that there was a continuous dense granular with particle sizes ranging from submicron to several micrometers. Some small voids can be found in these morphologies, and no obvious cracks were found. Figure 3 presents the initial cross-sectional morphology of the K6509 aluminum coating and the corresponding EDS line-scan and mapping results. The aluminum coating consists primarily of an outer layer and an interdiffusion zone (IDZ) [42], with a total thickness of approximately 20 μm. Under backscatter mode, the outer layer appears dark contrast and is primarily composed of Co, Ni, and Al, whereas the IDZ (~5 μm thick) exhibits bright contrast due to higher concentrations of Cr (red arrow and dashed line) and W. Line-scan analysis revealed a Cr concentration reaching 55%. Minor Cr-rich phases were also detected within the outer layer, indicating that interdiffusion occurs between the two regions and that the phase constituents are not strictly confined to their respective zones.

**Figure 4:**
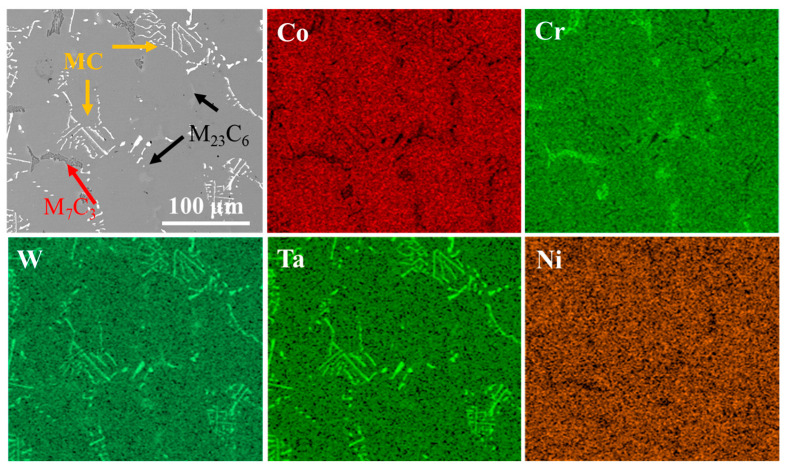
BSE morphology of the initial Co-based superalloy K6509 substrate and corresponding EDS mapping results.

Figure 4 presents the morphology and elemental mapping of Co-based superalloy K6509 substrate. The alloy matrix contains multiple types of carbides, mainly Cr-rich M_23_C_6_; Cr-/Co-rich M_7_C_3_; and Ta-/W-rich MC phases [43], as indicated by black arrows, red arrows, and yellow arrows, respectively in Figure 4. The specific morphology of the carbides is shown in Figure 4. These carbides serve as the primary strengthening phases in the alloy due to their exceptional thermal stability [44], which significantly enhances the high-temperature service performance.

### 3.2. Corrosion Removal Test

**Figure 5:**
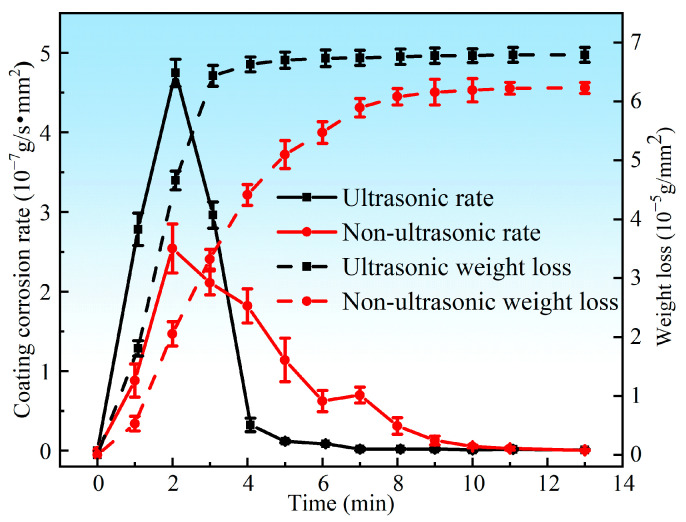
Corrosion rate and weight loss of the aluminum coating samples under ultrasonic and non-ultrasonic conditions.

**Figure 6:**
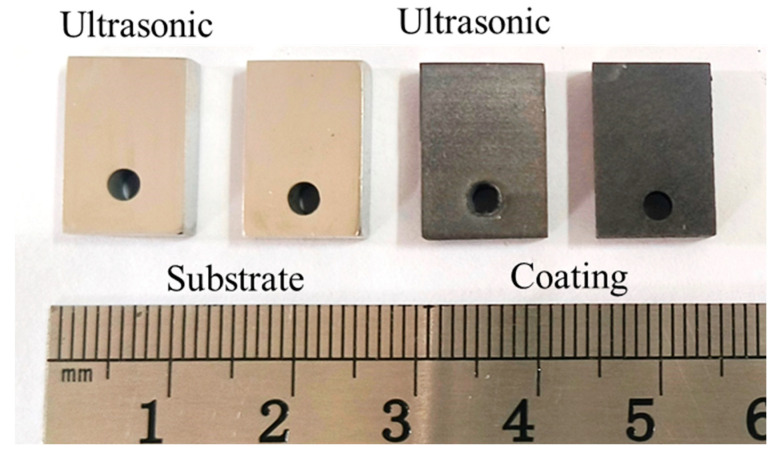
Macro-morphology of aluminum coating and substrate samples after corrosion under ultrasonic and non-ultrasonic conditions.

Figure 5 presents the corrosion rate and weight loss per unit area of the aluminum coating samples under ultrasonic and non-ultrasonic conditions. It is worth noting that the mass of the substrate remained essentially unchanged before and after corrosion (weight loss < 0.01 mg), so its corrosion curve was omitted. The results reveal that the corrosion rate increases initially, reaches a peak at 2 min, and then declines under both conditions. Under non-ultrasonic condition, the maximum corrosion rate is (2.5413 ± 0.3070) × 10^−7^ g·s^−1^·mm^−2^, with complete removal achieved at 10 min. Under ultrasonic, the rate is (4.7488 ± 0.1709) × 10^−7^ g·s^−1^·mm^−2^, and complete removal is achieved at 6 min. The final weight loss of the non-ultrasonic samples is (6.22841 ± 0.090481) × 10^−5^ g/mm^2^, compared to ultrasonic sample (6.79052 ± 0.126114) × 10^−5^ g/mm^2^. This difference suggests that some aluminum coating may remain adhered to the sample surface in the absence of ultrasonic, leading to incomplete removal. Figure 6 shows the macroscopic morphologies of the aluminum coating and substrate samples after corrosion under two conditions. The substrate maintained a bright, unaffected surface after corrosion. In the absence of ultrasonic treatment, the aluminum coating sample exhibited dark gray corrosion products, which could be easily detached. In contrast, the ultrasonic sample exhibited a uniform light gray surface, with sandblasted appearance after corrosion, indicative of more thorough and uniform coating removal.

**Figure 7:**
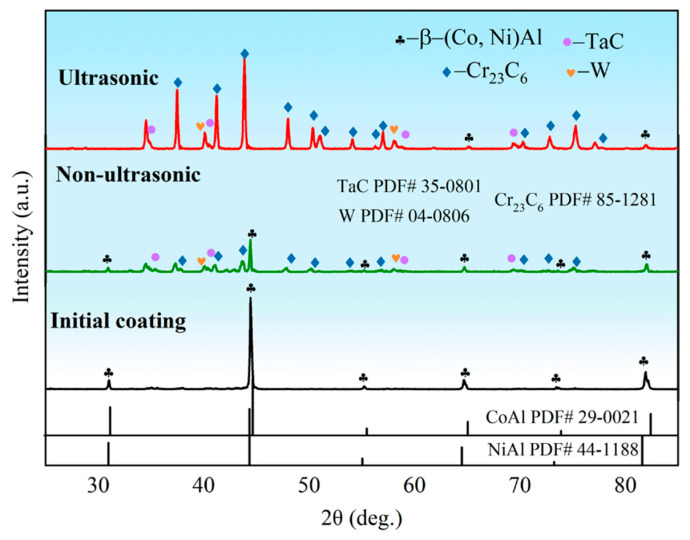
X-ray diffraction (XRD) pattern of the aluminum coating sample before corrosion and after corrosion removal under ultrasonic and non-ultrasonic conditions.

Figure 7 presents the X-ray diffraction (XRD) patterns of the initial coating sample and samples after corrosion under both ultrasonic and non-ultrasonic conditions. The initial coating primarily consisted of β-(Co,Ni)Al phase. After corrosion under ultrasonic, the corrosion front extended the interdiffusion zone (Figure 3). Weak peak intensity of β-(Co,Ni)Al was also observed, which is attributed to the residual trace amount of β-(Co,Ni)Al in the IDZ. Additionally, a large amount of Cr_23_C_6_, TaC, and W phases were identified. In contrast, the non-ultrasonic sample retained relatively stronger β-phase peaks while also containing Cr_23_C_6_, TaC, and W phases. The observed peak shifts in the non-ultrasonic sample may be attributed to increased surface roughness and corrosion-induced porosity.

**Figure 8:**
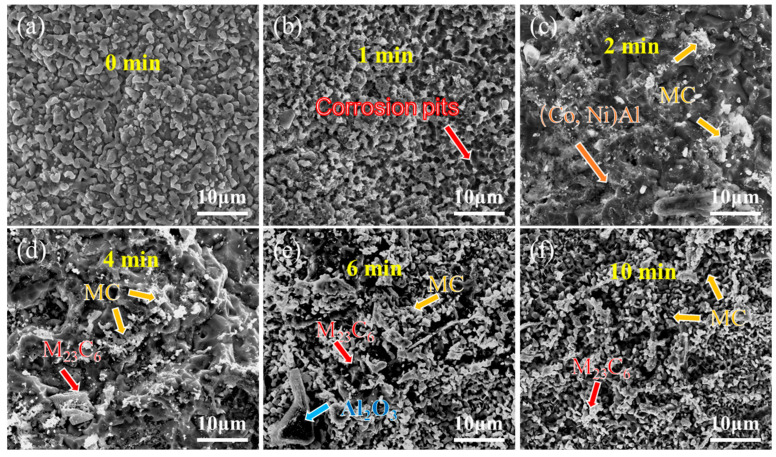
SEM morphology of aluminum coating samples under non-ultrasonic conditions after different corrosion durations: (**a**) 0 min; (**b**) 1 min; (**c**) 2 min; (**d**) 4 min; (**e**) 6 min; (**f**) 10 min.

**Figure 9:**
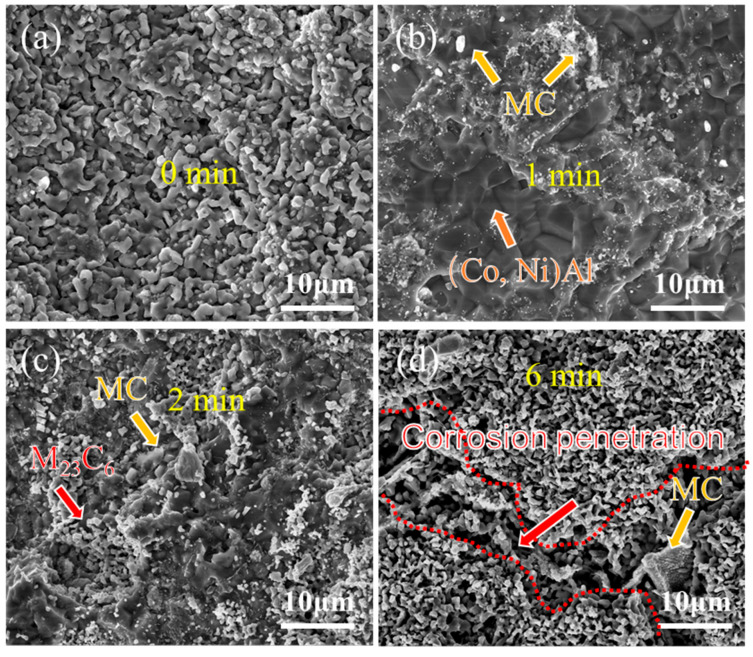
SEM morphology of aluminum coating samples under ultrasonic conditions after different corrosion durations: (**a**) 0 min; (**b**) 1 min; (**c**) 2 min; (**d**) 6 min.

**Figure 10:**
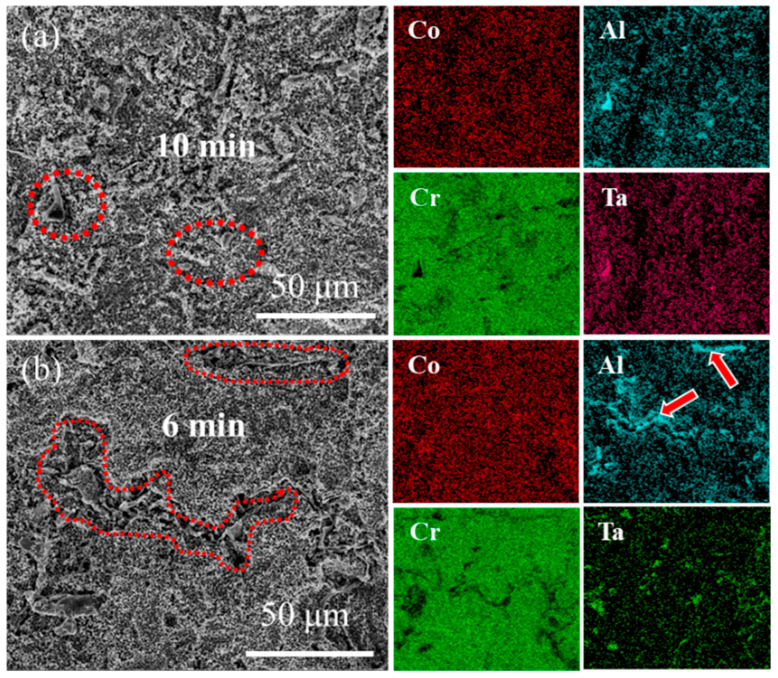
SEM morphology and corresponding EDS mapping of aluminum coating samples at the end of corrosion under (**a**) non-ultrasonic and (**b**) ultrasonic conditions.

Figure 8 and Figure 9 present the surface morphology evolution of aluminum coating samples at different corrosion times under non-ultrasonic and ultrasonic conditions, while Figure 10 presents the low-magnification morphology and corresponding mapping results after complete corrosion. Under non-ultrasonic conditions, the surface retained granular features after 1 min of corrosion (Figure 8b), though with indistinct grain boundaries and localized corrosion pits. At 2 min, sparse clusters of MC carbides emerged, indicating the presence of minor MC phases in the coating’s outer layer. Regular-shaped M_23_C_6_ carbides appeared at 4 min, and the amount of MC increased. After 6 min, abundant M_23_C_6_ carbides and micron-sized alumina particles were observed. Under ultrasonic treatment, the initial coating morphology was completely altered within 1 min, and more M_23_C_6_ and MC carbides formed by 2 min. Final corrosion morphology revealed corrosion trenches in specific regions (Figure 9d), with EDS confirming Al enrichment (Figure 10b, red arrows and dashed lines). Comparative analysis of the final surface morphologies under different conditions revealed distinct differences: the surface of non-ultrasonic sample showed significant roughness with substantial residual deposits (Figure 10a), whereas ultrasonic treatment yielded a smoother surface.

**Figure 11:**
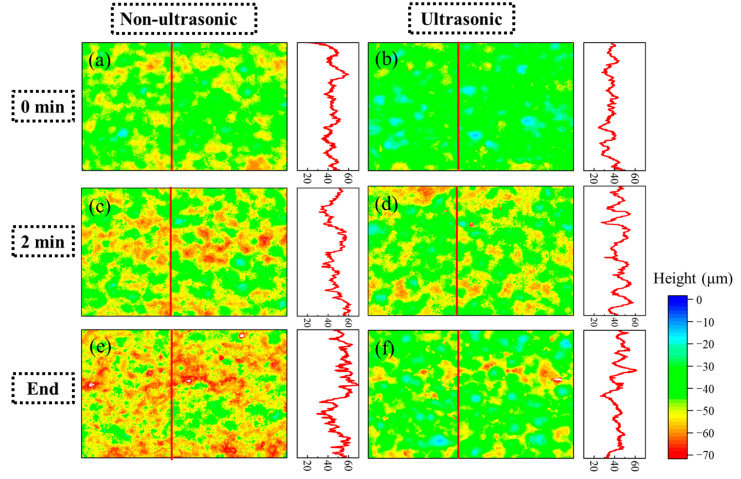
Laser spectroscopy confocal microscope profiles of the aluminum coating samples under ultrasonic (**b**,**d**,**f**) and non-ultrasonic (**a**–**c**) conditions corroded for different times: (**a**,**b**) 0 min; (**c**,**d**) 2 min; (**e**,**f**) 10/6min. Right panel: corresponding linear roughness profile marked by the red line in (**a**–**f**).

**Figure 12:**
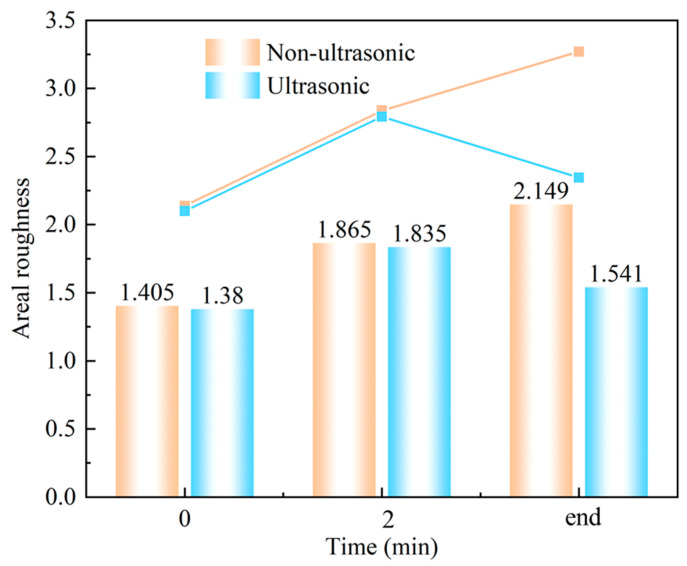
Laser spectroscopy confocal areal roughness mapping results of samples corroded for different times under ultrasonic and non-ultrasonic conditions.

Figure 11 shows laser confocal morphology of the samples at 0 min, 2 min, and the end of corrosion under both ultrasonic and non-ultrasonic conditions. The roughness of the corresponding to the red line is shown in the figure on the right. The corresponding surface roughness values are quantitatively compared in the histogram shown in Figure 12. The initial coating sample exhibited minimal height variation and relatively uniform surface morphology (Figure 11a,b). After 2 min of corrosion (Figure 11c,d), significant surface fluctuations developed due to vigorous corrosion reactions, leading to increased surface roughness. Notably, the non-ultrasonic treatment resulted in greater surface heterogeneity compared to ultrasonic conditions. After corrosion was finished, the non-ultrasonic sample (Figure 11e) demonstrated even more roughness enhancement because of the buildup of deposits and holes caused by corrosion. In contrast, ultrasonic corrosion (Figure 11f) terminated at the interdiffusion zone, yielding a more uniform surface with reduced roughness.

**Figure 13:**
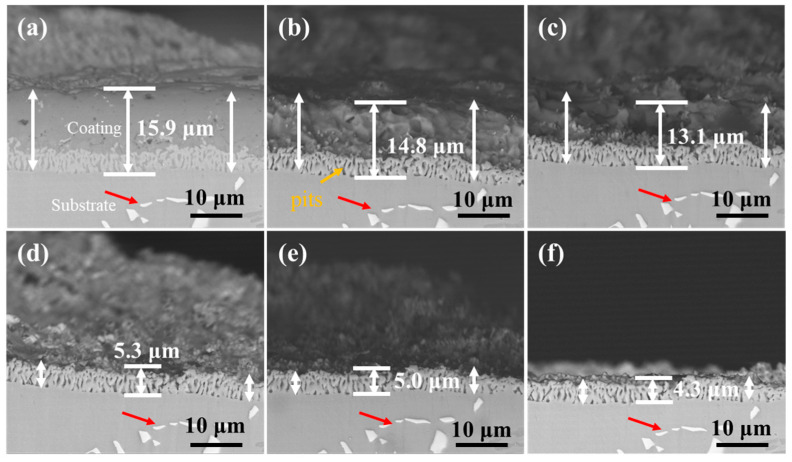
Semi-in situ observations of aluminum coating samples under non-ultrasonic conditions after different corrosion durations: (**a**) 0 min; (**b**) 1 min; (**c**) 2 min; (**d**) 4 min; (**e**) 6 min; (**f**) 10 min.

**Figure 14:**
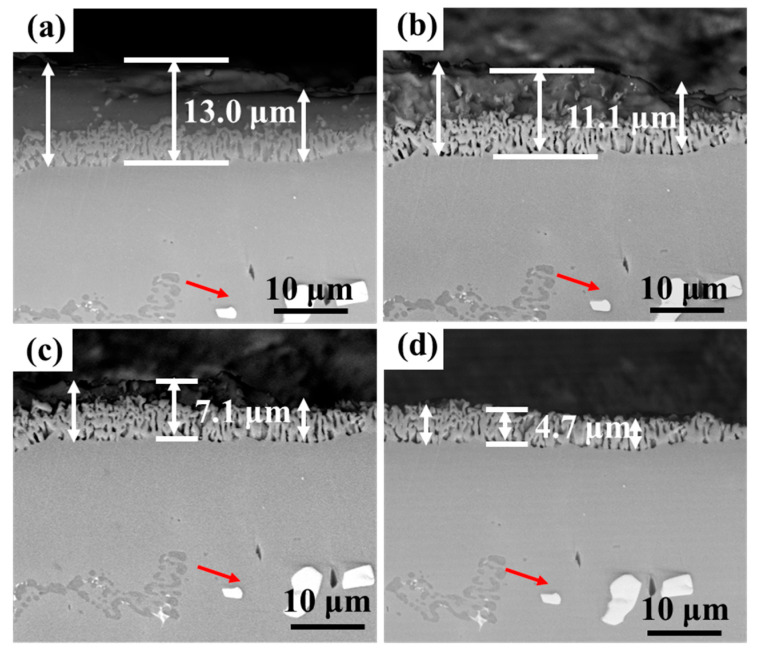
Semi-in situ observations of aluminum coating samples corroded under ultrasonic conditions after different corrosion durations: (**a**) 0 min; (**b**) 1 min; (**c**) 2 min; (**d**) 6 min.

Figure 13 and Figure 14 show the cross-section morphology of the coatings corroded for different times under ultrasonic and non-ultrasonic conditions, respectively. Semi-in situ observations reveal that the aluminum coating was gradually removed from the outer to the inner region (the coating thickness was determined via a semi-in situ method, with three measurements taken at different regions and the average value calculated). Simultaneously, Al-rich areas within the IDZ preferentially corroded (Figure 13b), leading to localized porosity. After 2 min of corrosion, the coating thickness decreased from 16.4 μm to 12.1 μm (26.22% reduction) under non-ultrasonic conditions. In contrast, ultrasonic treatment led to a more significant reduction from 14.2 μm to 7.8 μm, corresponding to 45.07% reduction. At the end of corrosion, an IDZ of 4.3 μm (non-ultrasonic) and 3.9 μm (ultrasonic) remained. Semi in situ observations confirmed that both the substrate microstructure and carbide morphology (red arrow) remained essentially unchanged throughout the corrosion process, demonstrating successful coating removal without damaging the substrate.

**Figure 15:**
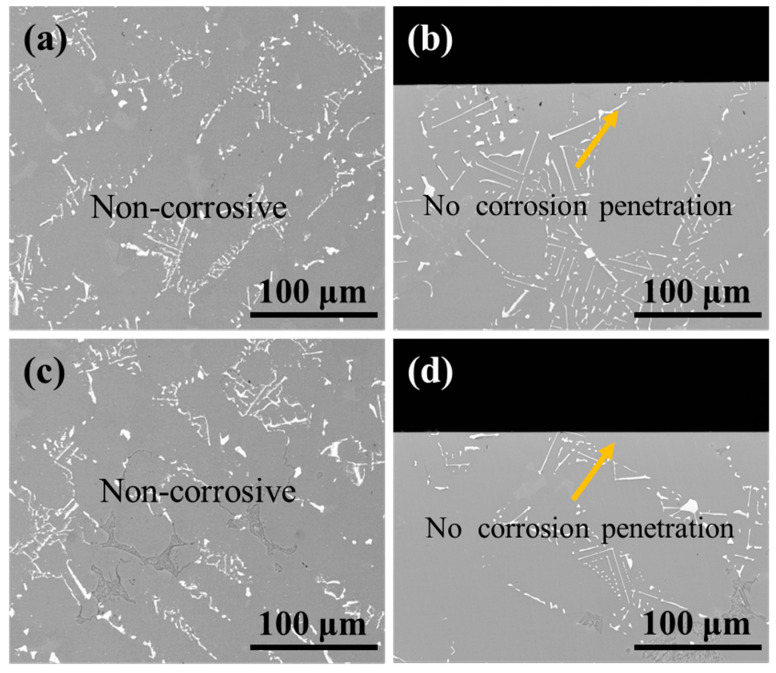
BSE images of (**a**,**c**) the surface and (**b**,**d**) cross-section morphology of substrate samples after corrosion under ultrasonic and non-ultrasonic conditions. (**a**,**b**) Non-ultrasonic; (**c**,**d**) ultrasonic.

As shown in Figure 15, the surface and cross-section morphology of the substrate samples revealed no detectable corrosion features or penetration after treatment under identical conditions. These results demonstrate that the 10% HNO_3_ solution effectively preserves substrate integrity regardless of ultrasonic application, thereby confirming the viability of this processing method.

### 3.3. Electrochemical Test

**Figure 16:**
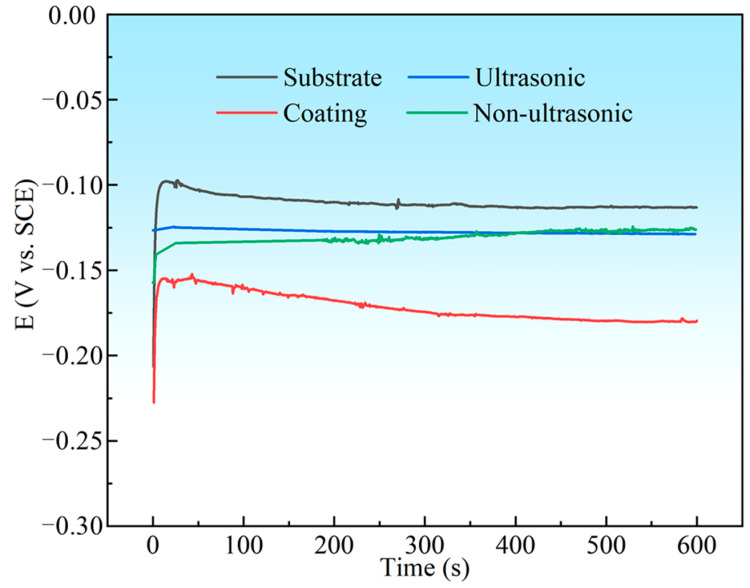
The open circuit potential (OCP) curves for substrate, initial coating, and coating samples after corrosion under ultrasonic and non-ultrasonic in 10% HNO_3_ solution.

Figure 16 summarizes the OCP values of the substrate, initial coating, and the samples after corrosion under ultrasonic and non-ultrasonic conditions during 600 s of immersion in 10% HNO_3_. The OCP of all samples shifted negatively at the initial stage of the immersion and finally stabilized at a value. The substrate exhibited the highest OCP in 10% HNO_3_ (−113 mV vs. SCE), while the initial coating showed the lowest OCP value (−179 mV vs. SCE) and struggled to reach a steady state, likely due to continuous surface–electrolyte interactions [45]. The samples with the coating removed (both under ultrasonic and non-ultrasonic conditions) exhibited intermediate potential between the substrate and the initial coating.

**Figure 17:**
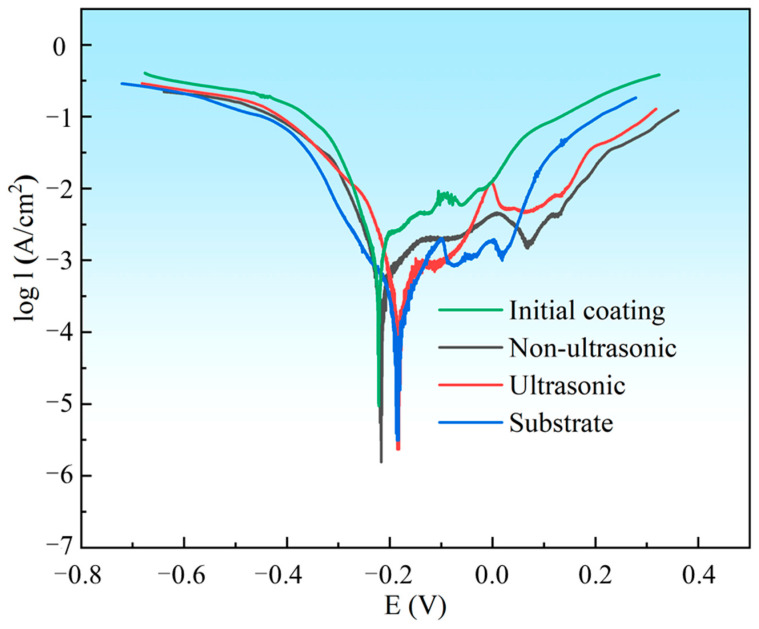
Potentiodynamic polarization (PDP) curves for substrate, initial coating, and the samples after corrosion under ultrasonic and non-ultrasonic conditions in 10% HNO_3_ solution. The PDP curves were scanned from −0.5 V to + 0.5 V vs. OCP with a scanning rate of 5 mV/s.

The PDP curves of the substrate, initial coating, and the samples after corrosion under ultrasonic and non-ultrasonic conditions in 10% HNO_3_ solution are shown in Figure 17. All samples except the initial coating exhibited stable passive regions, indicating that the coating can be completely dissolved in 10% HNO_3_ without forming a protective passive film. The corrosion potentials (*E_corr_*) and corrosion current densities (*I_corr_*) determined by Tafel extrapolation are summarized in Table 2. The substrate (−0.1830 ± 0.0040 V) and the samples after ultrasonic corrosion show higher *E_corr_* compared to the initial coating (−0.2241 ± 0.0013V) and the samples after non-ultrasonic corrosion. This indicates that they have a lower corrosion tendency in 10% HNO_3_ solution. Moreover, the *I_corr_* of the initial coating ((1.531 ± 0.110) × 10^−3^ A/cm^2^) is much higher than that of the substrate ((1.95 ± 0.13) × 10^−4^ A/cm^2^), while the *I_corr_* of the corroded samples is reduced compared to the initial coating. It shows that in 10% HNO_3_, the substrate exhibits significantly higher corrosion resistance than the initial coating, confirming that this solution system effectively removes the coating while minimizing damaging the substrate.

**Figure 18:**
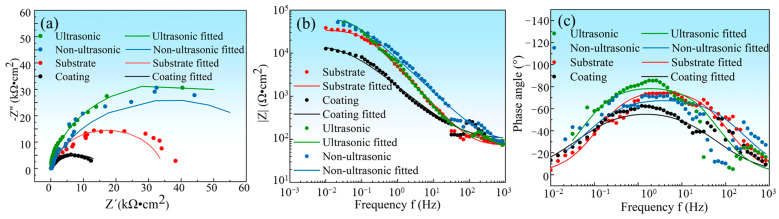
(**a**) The electrochemical impedance spectroscopy (EIS) plot of substrate, initial coating, and the samples after corrosion under ultrasonic and non-ultrasonic conditions in 10% HNO_3_ solution. (**a**) Nyquist plots, (**b**) Bode impedance magnitude plots, and (**c**) Bode phase angle plots; the EIS measurements were performed at OCP with frequency ranging from 10^4^ Hz to 0.01 Hz at a sinusoidal signal with an amplitude of 7 mV.

**Figure 19:**
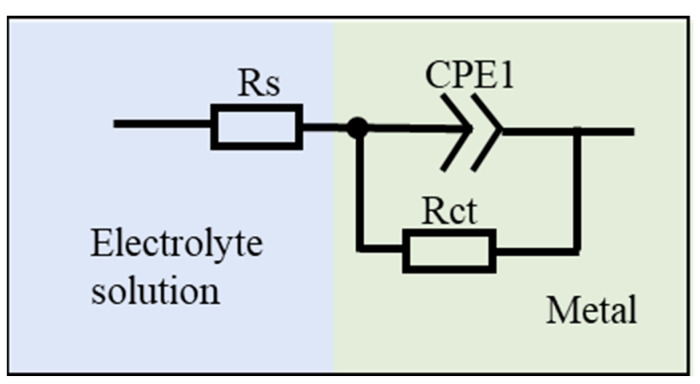
Equivalent circuit models proposed for the fitting of EIS spectra.

In order to investigate the corrosion mechanism of aluminum coating samples, electrochemical impedance spectroscopy (EIS) measurements were performed. Figure 18 presents the EIS results of substrate and coating samples before and after corrosion in 10% HNO_3_. The Nyquist plots show only one capacitive loop in the entire frequency range for all four samples, where the radius of the capacitive arc is related to the polarization resistance [46]. As polarization resistance directly reflects corrosion resistance [47], the smallest radius of the capacitive loop observed for the initial coating indicates its poorest corrosion resistance in 10% HNO_3_, which is consistent with the results of the polarization curve measurements. Corroded samples showed larger arc radius than the initial coating. The EIS data were fitted using the equivalent circuit model in Figure 19, where *R_s_* represents solution resistance, *R_ct_* is the charge transfer resistance, and constant phase element (CPE) is used to characterize the non-ideal capacitive behavior of coatings or passive films. Table 3 summarizes the fitting parameters of the EIS date. The initial coating sample has the lowest *R_ct_* value of (1.650 ± 0.232) × 10^4^ Ω·cm^−2^, while the sample corroded under ultrasonic conditions shows the highest *R_ct_* value of (7.395 ± 1.017) × 10^4^ Ω·cm^−2^. This indicates that after removing the outer layer of the coating, the IDZ possesses superior corrosion resistance compared to both the substrate material and the initial aluminum coating sample.

### 3.4. AFM and SKPFM Analysis

**Figure 20:**
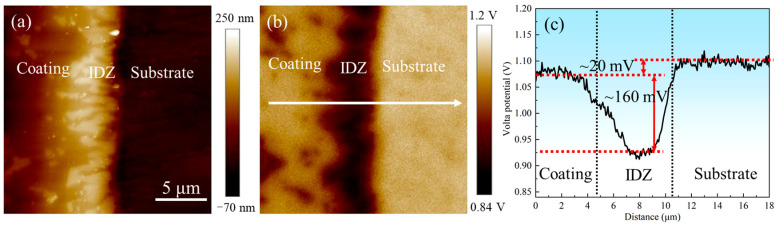
(**a**) Atomic force microscopy (AFM) and (**b**) scanning Kelvin probe force microscopy (SKPFM) photographs of the coating–substrate interface; (**c**) Volta potential variations along the line profile in (**b**).

AFM and SKPFM were employed to characterize the topography and electrical surface potential distribution across the outer layer, IDZ, and substrate, as presented in Figure 20. Both results revealed distinct interfacial boundaries among these regions, with the IDZ exhibiting the highest relative height and lowest Volta potential. The line profile in Figure 20c demonstrated that the substrate exhibits a surface potential approximately 20 mV higher than that of the coating, whereas the IDZ displays a surface potential approximately 160 mV lower, suggesting stronger thermodynamic driving force for corrosion in the IDZ. However, corrosion removal tests in 10% HNO_3_ revealed the IDZ exhibited significantly lower actual corrosion rates compared to the outer layer. This apparent contradiction highlights the limitations of interpreting corrosion behavior based solely on electrochemical potential. In practice, the corrosion response is governed not only by thermodynamic driving forces but also by the synergistic effects of phase composition, chemical stability, microstructure, and dynamic environmental factors [48]. In this case, the chemically stable Cr_23_C_6_ carbides within the IDZ imparts a strong resistance to acid attack, likely due to their passivation tendency [49] and low solubility in nitric acid. Therefore, despite having a lower surface potential, the IDZ demonstrates superior corrosion resistance compared to the outer β-(Co,Ni)Al-rich coating, illustrating the critical role of microstructural and compositional factors in governing localized corrosion behavior.

## 4. Discussion

### 4.1. Effect of Ultrasonic on the Corrosion

**Figure 21:**
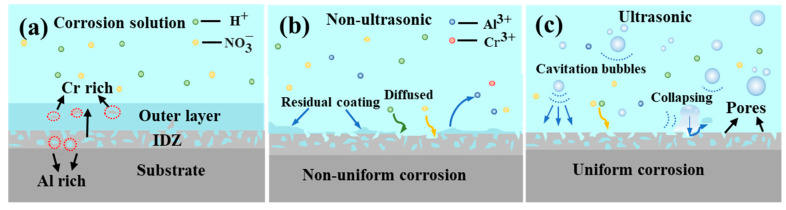
Corrosion mechanism diagram of K6509 coatings under different conditions. (**a**) Initial coating, (**b**) non-ultrasonic, (**c**) ultrasonic.

Figure 5 illustrates the effect of ultrasonic treatment on the coating removal rate. It is evident that ultrasonic treatment significantly enhanced the removal rate, promotes a more uniform removal process, and results in a smoother surface (Figure 11). The mechanism of the effect of ultrasonic on the removal of K6509 coating is presented in Figure 21. As shown in Figure 21a, with the sample immersed in 10% HNO_3_ solution, the initial coating structure consists of two layers: an outer layer predominantly composed of β-(Co,Ni)Al with minor Cr_23_C_6_ carbides, and an interdiffusion zone rich in Cr_23_C_6_ with residual β-(Co,Ni)Al and MC carbides. This microstructure is characteristic of low-temperature, high-activity aluminum coatings, where coating formation primarily occurs through inward aluminum diffusion, consistent with the findings reported by Shao et al. [50]. Under non-ultrasonic conditions (Figure 21b), the Al-rich β-(Co,Ni) Al phase is preferentially corroded (Figure 13b). In some regions, the presence of Cr_23_C_6_ slows down the corrosion rate, resulting in uneven coating corrosion. The corrosive solution continuously penetrates the coating through-holes created in the preferentially corroded areas, causing further corrosion. By the end of the corrosion process, the faster-corroding regions reach the IDZ, while in the slower-corroding regions, some parts of the aluminum coating remain unremoved due to the adhesion of corrosion products or the presence of refractory Cr_23_C_6_. Under ultrasonic conditions (Figure 21c), the effective and rapid removal of the aluminum coating is primarily achieved through cavitation effects and enhanced ion diffusion. Cavitation effects mainly originate from bubbles generated during the chemical corrosion reaction of the coating and ultrasonic vibration. When bubbles approach the coating, the pressure and solution jets generated by bubble collapse impact the coating through two mechanisms: (i) accelerating the spall of the loose coating and the exposure of fresh surface [51,52], and (ii) enhancing the penetration of corrosive solution through existing pores to facilitate further corrosion. Furthermore, ultrasonic shockwaves promote ${\mathrm{NO}}_{3}^{-}$, Al^3+^, Co^2+^, and H^+^ diffusion, thereby accelerating chemical corrosion reactions. Subsequently, the synergistic effects of continuous microjet impact and corrosive ions in the solution [53] contribute to increased coating removal rates and a more uniform surface.

### 4.2. Corrosion Process of Aluminum Coating

The corrosion behavior of the aluminum coating on Co-based superalloy K6509 in 10% HNO_3_ initiates in the outer layer and progresses inward (Figure 13 and Figure 14), with no significant damage to the substrate observed (Figure 15). As shown in Table 2, the coating exhibits a higher corrosion tendency and a larger corrosion current density (approximately one order of magnitude) compared to the substrate in 10% HNO_3_. According to the ASTM G102 standard, the corrosion rate is directly proportional to the corrosion current density, as shown in Equation (3) [54]:

(3) $$v=\frac{i_{\mathrm{corr}}\cdot M}{n\cdot F\cdot \rho }$$

where v is the corrosion rate (g·m^−2^·h^−1^), $i_{corr}$ is corrosion current density (A·cm^−2^), *M* is the molar mass (g/mol), *n* is the number of electrons transferred in the corrosion reaction, *F* is Faraday’s constant (96,485 C/mol), and *ρ* is the material density (g/cm^3^). This confirms that the corrosion rate of the substrate is significantly lower than that of the coating.

AFM and SKPFM results revealed a 160 mV higher potential in the outer β-(Co,Ni)Al-rich layer compared to the IDZ containing Cr_23_C_6_, TaC, and W phases. Cross-sectional corrosion morphology (Figure 13 and Figure 14) demonstrated that corrosion initiated preferentially in the outer layer, with the darker β-phase in the IDZ corroding before the brighter Cr_23_C_6_ phase, forming porous structures. Remarkably, the corrosion rate approached zero upon reaching the Cr_23_C_6_-rich regions of the IDZ (Figure 5). Post-corrosion samples exhibited lower *I_corr_* and higher *R_ct_* compared to the initial coating (Table 3), confirming enhanced corrosion resistance at the IDZ. This improvement stems from the IDZ’s dominant Cr_23_C_6_ phase, which has a face-centered cubic (fcc) structure and a primitive unit cell that has 23 metal atoms occupying four non-equivalent metal sites while 6 carbon atoms occupy the Wyckoff 24e sites [55], resulting in extremely high chemical stability. In contrast, β-(Co,Ni)Al, as an intermetallic compound, contains numerous structural defects (such as dislocations and vacancies), which increase its susceptibility to localized corrosion [56]. Guével et al. [28]. used electrochemical methods to dissolve the aluminum coatings on pure Ni, Ni20Cr, and René 125 Ni-based superalloys. Their results showed that HNO_3_ promotes the dissolution of Al coating.

Additionally, the observed decrease in corrosion rate with increasing Cr content was attributed to the formation of a passive film, consistent with our experimental findings. The chemically inert Cr_23_C_6_ phase resists dissolution, and even when minor reactions occur, a dense and protective Cr_2_O_3_ film rapidly forms, further enhancing corrosion resistance. This corrosion behavior caused parts to corrode unevenly and created a rough surface, accounting for the observed increase in surface roughness after 2 min corrosion under both ultrasonic and non-ultrasonic conditions (Figure 12). As corrosion progresses into the IDZ, it encounters resistance to reaction because of its chemical stability. So, the IDZ helps slow down corrosion by preventing the corrosive material from spreading, which protects the underlying surface.

Compared with previous works, where HCl- or mixed-acid systems were typically used for aluminide coating removal on Ni-based alloys [28,35], the present study demonstrates that 10% HNO_3_ provides a more controlled removal route with reduced risk of pitting corrosion. Importantly, most earlier reports focused on Ni-based superalloys, whereas investigations on Co-based alloys remain scarce. Furthermore, purely chemical methods often suffer from prolonged treatment times and uneven removal [40]. By integrating ultrasonic treatment, our approach overcomes these drawbacks, thereby reducing the total removal time from 10 min to 6 min while achieving a smoother surface. The choice of 10% HNO_3_ represents a balance between efficiency and substrate protection, ensuring complete coating removal without inducing excessive damage. This combined chemical–ultrasonic strategy therefore provides a promising pathway for efficient and reliable coating removal in complex-shaped Co-based superalloy components, offering clear advantages for practical recoating and industrial remanufacturing applications.

## 5. Conclusions

This study systematically investigates the effects of ultrasonic treatment on the removal efficiency of aluminum coatings from the Co-based superalloys K6509, as well as the corrosion behavior of the coating in a 10% nitric acid solution system. The main conclusions are as follows:

Ultrasonic can significantly increase the coating removal rate. The maximum corrosion rate under ultrasonic conditions reached 4.7488 × 10^−7^ g·s^−1^·mm^−2^, which is 46.49% higher than that without ultrasonic (2.5413 × 10^−7^ g·s^−1^·mm^−2^). This improvement is primarily attributed to the cavitation effect of bubbles and the enhanced diffusion of solution ions driven by ultrasonic shock waves, which collectively promote faster and more uniform coating removal, resulting in a smoother surface.

The electrochemical impedance and potentiodynamic polarization spectroscopy measurements confirmed that the coating is significantly more susceptible to corrosion in 10% HNO_3_ than the underlying substrate. These results also demonstrate that the nitric acid system effectively removes the aluminum coating without compromising substrate integrity.

The corrosion initiates in the outer layer and progresses inward. The distinct corrosion behaviors, where β-(Co,Ni)Al dissolves in HNO_3_ while Cr_23_C_6_ resists dissolution due to its chemical stability and tendency to passivate, leads to localized and non-uniform corrosion. The interdiffusion zone, enriched with Cr_23_C_6_, served as an effective barrier that limits corrosive solution penetration and provides substantial protection to the substrate.

## Figures and Tables

**Table 1:** Nominal chemical composition of K6509 Co alloy (wt.%).

Cr	Ni	W	Ta	C	Zr	Ti	Co
23.0~24.0	9.0~11.0	6.5~7.5	3.0~4.0	0.55~0.65	0.3~0.6	0.15~0.25	Bal.

**Table 2:** The corrosion potential (*E_corr_*) and corrosion current density (*I_corr_*) measured from the polarization curves for substrate, initial coating, and coating samples corroded under ultrasonic and non-ultrasonic conditions.

		*E_corr_* (V)	*I_corr_* (A/cm^2^)
Before corrosion	Substrate	−0.1830 ± 0.0040	(1.95 ± 0.13) × 10^−4^
	Initial coating	−0.2241 ± 0.0013	(1.531 ± 0.110) × 10^−3^
After corrosion	Ultrasonic	−0.1826 ± 0.0028	(1.57 ± 0.43) × 10^−4^
	Non-ultrasonic	−0.2285 ± 0.0050	(4.734 ± 2.72) × 10^−4^

**Table 3:** Fitting parameters for the EIS data recorded of substrate, initial coating, and coating samples under ultrasonic and non-ultrasonic conditions in 10% HNO_3_ solution. *Rs*: the solution resistance; *R_ct_*: the polarization resistance; *CPE-T*, *CPE-P*: the CPE constant.

Samples	*R_s_* (Ω·cm^−2^)	*CPE-T*/10^−5^ (F·cm^−2^)	*CPE-P* (F·cm^−2^)	*R_ct_*/10^4^ (Ω·cm^−2^)
Substrate	71.8 ± 2.989	3.963 ± 0.150	0.888 ± 0.0099	3.457 ± 0.174
Initial coating	100.8 ± 7.356	16.14 ± 0.993	0.695 ± 0.0190	1.650 ± 0.232
Ultrasonic	95.15 ± 4.578	3.900 ± 0.171	0.914 ± 0.0138	7.395 ± 1.017
Non-ultrasonic	89.42 ± 7.865	3.138 ± 0.204	0.789 ± 0.0159	7.278 ± 1.163

## Data Availability

The original contributions presented in this study are included in the article. Further inquiries can be directed to the corresponding author.
